# Rheological Properties of Sodium Carboxymethylcellulose Solutions in Dihydroxy Alcohol/Water Mixtures

**DOI:** 10.3390/ma16010418

**Published:** 2023-01-02

**Authors:** Patrycja Wagner, Sylwia Różańska, Ewelina Warmbier, Adrianna Frankiewicz, Jacek Różański

**Affiliations:** Department of Chemical Engineering and Equipment, Faculty of Chemical Technology, Poznan University of Technology, ul. Berdychowo 4, PL 60-965 Poznan, Poland

**Keywords:** sodium carboxymethylcellulose, polyhydric alcohol, rheology

## Abstract

The aim of the research presented in this paper was to determine the effect of dihydroxy alcohols on the rheological properties of sodium carboxymethylcellulose (Na-CMC) solutions with different degrees of substitution and different average molecular masses. Rheological measurements were carried out with a rotational rheometer in continuous and oscillatory flows. Two dihydroxy alcohols were used in the study: butane-1,3-diol and propane-1,2-diol. The concentration of Na-CMC in the solutions was 1.6% and 2.2%, while the concentration of the dihydroxy alcohols ranged from 10% to 60%. The measurements show that the viscoelastic properties of Na-CMC solutions are strongly linked to the type of solvent used. The application of low-substituted high-molecular-mass Na-CMC makes it possible to obtain fluids with the properties of weak physical gels. On the other hand, the dissolution of Na-CMC with a high degree of substitution (>1) and low molecular mass in dihydroxy alcohol/water mixtures yields a viscoelastic fluid. Based on oscillatory measurements, increasing concentrations of polyhydroxy alcohols in Na-CMC solutions were found to induce an increase in the strength of the network structure. At the same concentrations of polyhydroxy alcohols in solutions containing butane-1,3-diol, a stronger network structure is formed compared to solutions containing propane-1,2-diol. The rheological measurement results presented in this paper may be useful in the formulation of drug carriers and cosmetics in which rheological properties are a significant factor.

## 1. Introduction

Sodium carboxymethylcellulose (Na-CMC) is the only cellulose derivative included within the group of polyelectrolytes [1]. Based on its properties, mainly the ability to contribute to the desired consistency of products, sodium carboxymethylcellulose has found its way into a range of industrial sectors, with applications mainly in the production of food, cosmetics, and pharmaceuticals [2,3,4]. The global market for products (foodstuffs, beverages, cosmetics, medicines, and detergents) containing Na-CMC is huge (in 2016, the world market was worth USD 1.2 billion) [5]. Sodium carboxymethylcellulose is also used in large quantities in the oil and gas industry for thickening purposes in drilling muds [6,7]. In the food industry, Na-CMC is used, among other applications, to improve the moisture content of products and to give them the desired consistency while preventing the separation of ingredients [8]. In the cosmetics industry, Na-CMC is added to toothpastes and creams as a thickening agent [3,9]. As regards pharmaceuticals, Na-CMC is employed in the production of hydrogels that are used in biomedical engineering as components of drug delivery matrices (carriers for the controlled release of active substances in medicines) [10,11,12,13]. Commercial products contain a vast range of ingredients (silicas, oils, surfactants, proteins, polyols, sugars, and salts) that may interact with Na-CMC, resulting in new substances characterized by specific properties that differ from those of single-component solutions. For the reasons mentioned above, studies investigating the effects of various substances, including polyhydroxy alcohols, on the rheological properties of Na-CMC solutions carry a great practical significance.

Aqueous solutions of Na-CMC are non-Newtonian shear thinning fluids (an explanation of basic rheological concepts such as shear thinning fluids and others can be found in the paper of Ahmad et al. [14]). Research findings published to date show that the rheological properties of aqueous Na-CMC solutions depend not only on the concentration and molecular mass of the polymer but also on its degree of substitution (DS; average number of carboxymethyl groups substituted per anhydroglucose at the 2-, 3-, and 6-positions) [15,16,17,18,19,20,21,22,23,24,25,26,27]. The effects of monovalent and divalent salts on the rheological properties of Na-CMC solutions are also relatively well understood [28,29,30,31,32]. It must be noted that while the direction of changes in the viscoelastic properties of Na-CMC solutions occurring along with changes in DS is predictable, the reasons for these changes proposed in the literature to date have not been fully validated. Various authors have linked them to electrostatic interactions, hydrogen bonding, and hydrophobic interactions between unsubstituted parts of the chain [24,33,34,35].

Sodium carboxymethylcellulose and polyhydroxy alcohol are compounds that may coexist in many products. It appears that the rheological properties of solutions of Na-CMC/polyhydroxy alcohol mixtures may diverge significantly from those of single-component solutions of these compounds [22]. However, the properties of Na-CMC solutions in polyhydroxy alcohol/water mixtures have been described only in a few publications to date [22,36,37,38,39,40,41].

The results of measurements for Na-CMC solutions in a propane-1,2-diol/water mixture (PG/water) are presented in the works of Matsumoto and Mashiko [38], Komorowska et al. [22], and Różańska et al. [39]. Matsumoto and Mashiko [38] found that the superposition principle applies to Na-CMC solutions in mixtures containing different concentrations of PG, CaCl_2_ (calcium chloride), and MgCl_2_ (magnesium chloride). The cumulative curve was determined for PG at concentrations of 30% and 40% and Na-CMC with DS = 1.13 and M_w_ = 2.22 × 10^5^ g·mol^−1^. Komorowska et al. [22] conducted continuous and oscillatory flow measurements for Na-CMC solutions in a propane-1,2-diol/water mixture with three different degrees of polymer substitution (0.62, 0.79, and 1.04) and a similar average molecular mass (M_w_ = ~250,000 g·mol^−1^). The authors observed a strong synergism between the molecules of Na-CMC with a low degree of substitution and propane-1,2-diol, which they attributed to the formation of a network of a physical nature. Różańska et al. [39] carried out extensional flow measurements for solutions of Na-CMC in the DS range from 0.62 to 1.04 and with M_w_ = ~250,000 g·mol^−1^ in a propane-1,2-diol/water mixture. The results of these measurements point towards the formation of a spatial network of a physical nature.

Yang and Zhu [41] conducted studies for Na-CMC solutions with added propane-1,2,3-triol, concluding that the addition of polyhydroxy alcohol up to a concentration of 70% led to an increase in the viscosity of the Na-CMC solutions (there are no data on DS and M_w_). At higher concentrations of glycerol, the viscosity of the Na-CMC solutions decreased, which was attributed by the authors of the study to a decrease in the solubility of the polyelectrolyte. The sharp increase in viscosity caused by the presence of propane-1,2,3-triol was explained by physical cross-linking due to the formation of hydrogen bonds between the OH groups of the alcohol and the polymer chains.

Jimenez et al. [42] carried out research on the rheological properties of Na-CMC solutions in a glycerol/water mixture in extensional and shear flow. These authors showed that the addition of glycerol to the Na-CMC solutions increases the shear viscosity, extensional viscosity, and extensional relaxation time of formulations. However, the concentration-dependent variation of rheological properties cannot be simply modeled by accounting for the change in solvent viscosity and dielectric constant.

An increase in viscosity due to the addition of sucrose to Na-CMC solutions has been reported by Hoefler [37] and Cancela et al. [36]. However, the studies by these authors lack information on the degree of substitution and average molecular mass of the Na-CMC used. Cancela et al. [36] described the flow curves for Na-CMC solutions with added sucrose using the Ostwald–de Waele power model. In their paper, Sharma et al. [40] described the effect of ethylene glycol concentrations in the range of 10–30% on the intrinsic viscosity of Na-CMC solutions. Their studies were carried out for sodium carboxymethylcellulose with a relatively low average molecular mass (M_w_ = 90,000 g·mol^−1^) and DS = 0.7. Increasing ethylene glycol concentrations were found to be accompanied by decreases in intrinsic viscosity and increasing values of the Huggins constant. 

As can be seen from the studies presented above, data on the effects of polyhydroxy alcohols on the properties of Na-CMC solutions are scarce. A review of the literature reveals that there are no published studies on Na-CMC solutions with the addition of butane-1,3-diol. Furthermore, there are no literature reports on the impact of the molecular mass of Na-CMC on the rheological properties of the solutions of this polyelectrolyte in a propane-1,2-diol/water mixture.

The aim of the work presented in this paper was to determine the effect of the degree of substitution and average molecular mass on the rheological properties of Na-CMC solutions in mixtures of propane-1,2-diol (propylene glycol, PG) and butane-1,3-diol (butylene glycol, BG). Na-CMC solutions in such mixtures—similarly to the solutions of other polysaccharides—can potentially be used as drug delivery systems because of their high viscosity, which can be adjusted as needed through an appropriate selection of ingredients and their concentrations [43,44]. The findings of the present study can also translate into practical applications in the formulation of products in the food and cosmetics industries.

## 2. Materials and Methods

### 2.1. Materials

The sodium salt of carboxymethylcellulose with two different degrees of substitution, 0.7 and 1.2 (marked with the following symbols: Na-CMC_0.7-L_ and Na-CMC_1.2_), and similar weight-average molecular weights was used for the study. Na-CMC_0.7-L_ and Na-CMC_1.2_ had molecular masses of 264,400 g·mol^−1^ and 262,400 g·mol^−1^, respectively (supplier: Sigma-Aldrich).

In the conducted research, sodium carboxymethylcellulose with a much higher weight-average molecular weight of M_w_ = 1,206,000 g·mol^−1^ and a degree of substitution of 0.7 (supplier: Dow Chemical Company, Midland, United States) was also used. In the further part of the work, solutions using this polyelectrolyte will be marked with the symbol Na-CMC_0.7-H_. The molar mass distributions of Na-CMC samples were characterized by size-exclusion chromatography (SEC) with triple detection on the chromatograph composed of a Knauer K-501 HPLC pump (Berlin, Germany) with an LDC RI detector and a Viscotek T60A dual detector (right-angle laser light scattering at k = 670 nm (RALLS) and differential viscometer) (Malvern Panalytical Ltd, Malvern, United Kingdom).

The tests of mixtures of aqueous solutions of Na-CMC with polyhydric alcohols were carried out using propane-1,2-diol (propylene glycol, PG) and butane-1,3-diol (butylene glycol, BG), supplied by Dow Chemical Company, Donauch, and Sigma Aldrich. The weight content of the polyhydric alcohols used was ≥99.5%.

### 2.2. Preparations of Solutions

In order to obtain aqueous Na-CMC solutions at specific percentage concentrations (wt%), the polymer was gradually added to a beaker containing a measured amount of water. The aqueous Na-CMC solutions were stirred for approximately 24 h. After this time, the solutions were placed in a refrigerator and stored at 4°C. To prevent biological degradation, measurements with aqueous Na-CMC solutions were carried out within a maximum of 7 days from sample preparation.

**Figure 1:**
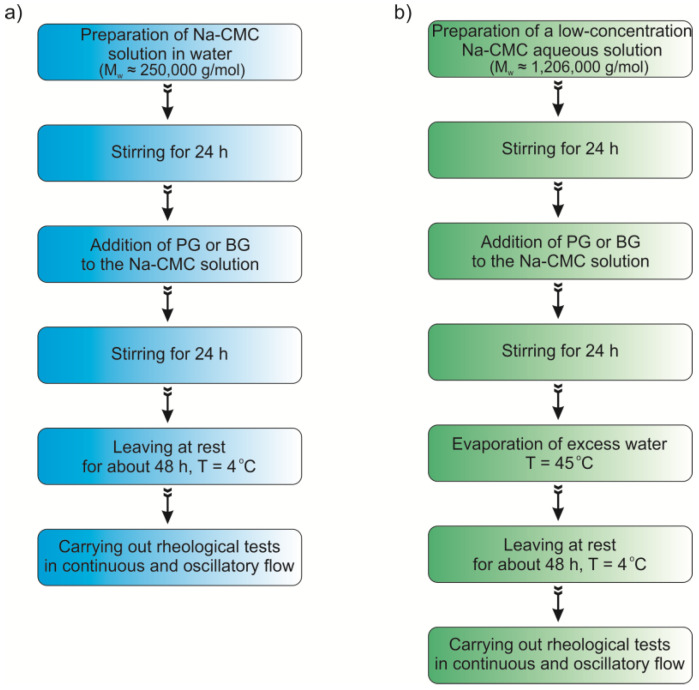
Flowchart for the preparation of Na-CMC solutions with a weight-average molecular weight of (**a**) 250,000 g/mol (Na-CMC_0.7-L_ and Na-CMC_1.2_) and (**b**) 1,206,000 g/mol (Na-CMC_0.7-H_).

Low-molecular-mass Na-CMC solutions (M_w_ = ~2.5 × 10^5^ g∙mol^−1^) in a polyhydroxy alcohol/water mixture were prepared at room temperature. An appropriate amount of Na-CMC was slowly added to a beaker containing a measured amount of water. In the next step, the samples were mixed using a magnetic stirrer until the polymer was completely dissolved. After 24 h, an appropriate dihydroxy alcohol was added in portions while stirring the solutions continuously. Between the tests, all the samples were stored at 4 °C. The solution preparation procedure is shown schematically in Figure 1.

Solutions containing Na-CMC with M_w_ = 1,206,000 g·mol^−1^ (Na-CMC_0.7-H_) required a modified preparation procedure to ensure that homogeneous solutions would be obtained. In this case, the procedure described above was used for the preliminary preparation of a solution with a lower concentration of Na-CMC and polyhydroxy alcohol than required (Na-CMC was dissolved in a larger volume of water). After preparing the lower-concentration solution, it was heated to 45 °C to evaporate excess water. Higher temperatures were undesirable because of the risk of thermal degradation of the polymer. The samples were occasionally weighed to make sure that correct concentrations of Na-CMC and alcohol were achieved. All the solutions obtained via this procedure were stored in a refrigerator at 4 °C.

### 2.3. Rheological Measurements

Rheological measurements were carried out using a Physica MCR 501 rotational rheometer (Anton Paar, Graz, Austria) in continuous and oscillatory flow at 20 °C. Continuous flow measurements were performed in the shear rate range from 0.01 s^−1^ to 1000 s^−1^. In addition, curves of instantaneous shear stresses *τ*^+^ versus strain γ (γ=φ/tan(α), where *α* is the cone angle and *φ* is the deflection angle) were recorded at a constant shear rate $\dot{\gamma }$ of 0.1 s^−1^ using a cone–plate measurement system (diameter = 59.974 mm; cone angle α = 2.014^°^).

The oscillatory measurements were carried out in a plate–plate system in the range of oscillation frequency from 0.01 rad/s to 100 rad/s. The plate diameter was 59.972 mm and the gap width was 1 mm.

The oscillatory measurements were made in a range of linear viscoelasticity with a value of the strain amplitude equal to 1%. The range of linear viscoelasticity was determined by measuring the values of the G′ and G″ modules at increasing strain amplitude from 0.01% to 1000% and a constant frequency of 1 Hz.

## 3. Results

### 3.1. Effect of the Degree of Substitution

**Figure 2:**
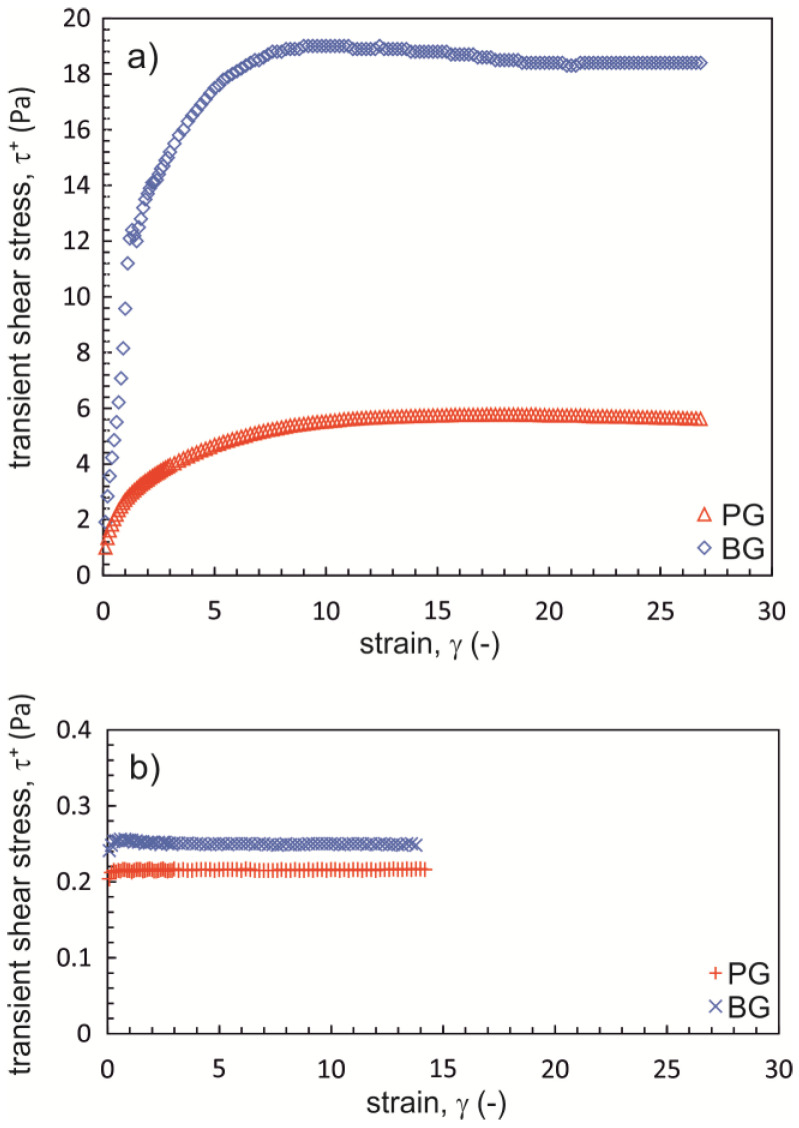
Comparison of the relationship *τ*^+^ = *f*(*γ*) for Na-CMC solutions with (**a**) DS = 0.7 and (**b**) DS = 1.2 in a mixture of PG/water and BG/water (Na-CMC concentration = 2.2%; M_w_ = ~250,000 g·mol^−1^; PG (propane-1,2-diol) and BG (butane-1,3-diol) concentration = 50%; $\dot{\gamma }$ = 0.1 s^−1^; T = 20 °C).

The measurement results obtained for transient shear stress τ^+^ as a function of strain γ in Na-CMC solutions (2.2%) with different degrees of substitution (DS = 0.7 and 1.2) and a similar average molecular mass (M_w_ = ~250,000 g·mol^−1^) in PG and BG mixtures (50%) are shown in Figure 2. Three shapes can be distinguished for the curve *τ*^+^ = *f*(*γ*):

- Solution Na-CMC_1.2_: transient shear stress values do not depend on the values of strain;
- Solution Na-CMC_0.7-L_ with added PG: transient shear stress values increase until reaching a constant value at a certain level of strain;
- Solution Na-CMC_0.7-L_ with added BG: transient shear stress values increase until reaching a specific maximum level (τ^+^_max_) at a certain strain value (γ_max_), after which they decrease and reach a constant level.

The characteristic maximum transient value of shear stress on the curve *τ*^+^ = *f*(*γ*) is referred to as stress overshoot, and it is typical of polymer fluids in which a spatial network made up of macromolecule chains has been formed. Thus, the data in Figure 2 show that a network of this type was formed in the solution of Na-CMC_0.7-L_ in mixture with BG and partially formed in the solution of Na-CMC_0.7-L_ in a PG mixture. However, no such network was found in the solution of Na-CMC_1.2_ [19,45]. Despite having established that stress overshoot τ_max_ is characteristic of fluids in which a spatial network has been formed, the phenomenon is not fully explained at the molecular level [46].

**Figure 3:**
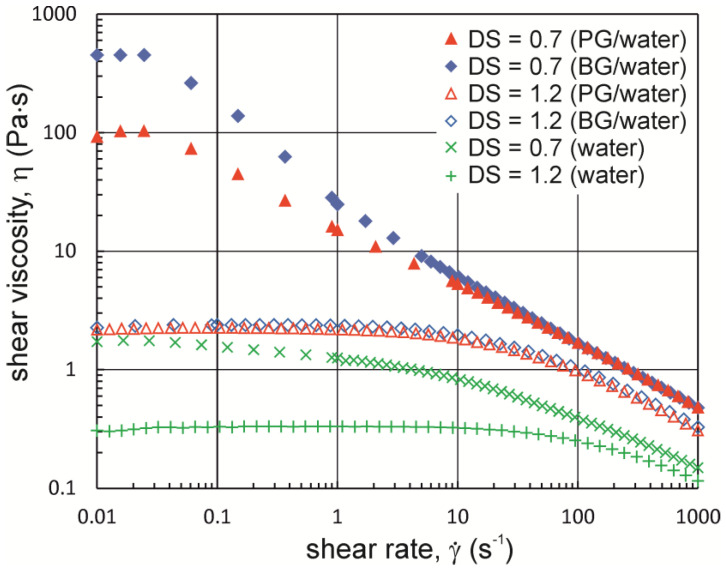
Viscosity curves for Na-CMC solutions (DS = 0.7 and 1.2) in distilled water and in PG/water and BG/water mixtures (Na-CMC concentration = 2.2%; M_w_ = ~250,000 g·mol^−1^; PG and BG concentration = 50%; T = 20 °C).

In the next experiment, viscosity curves were determined for Na-CMC solutions in water as well as PG/water and BG/water mixtures. The viscosity curves shown in Figure 3 were plotted based on the shear stress values measured after they had stabilized at a constant level (Figure 2). Based on the results obtained, it can be concluded that both a lower DS and the presence of dihydroxy alcohol affect the viscosity levels in Na-CMC solutions. In addition, the greatest differences occurred in the range of low shear rates. For better illustration of the differences, zero shear viscosity values *η*_0_ were determined by applying the Cross model to describe the viscosity curves (Table 1).

(1) $$\eta =\frac{\eta_{0}}{1+{\left(\lambda \cdot \dot{\gamma }\right)}^{m}}$$

where *λ* is the characteristic time, and *m* is the related exponent.

However, the Cross model was found to be unsuitable for describing the viscosity curves of Na-CMC with DS = 0.7 in the PG/water and BG/water mixtures. For these reasons, it was assumed in this case that zero viscosity was equal to the arithmetic mean calculated from the viscosities recorded in the shear rate range from 0.01 s^−1^ to 0.25 s^−1^ (the first three measurement points in Figure 3). 

A comparison of the zero viscosity values in aqueous solutions of Na-CMC_0.7-L_ and in polyhydroxy alcohol/water mixtures shows that the addition of butane-1,3-diol induces an approximately 240-fold increase in zero shear viscosity, while the addition of propane-1,2-diol results in an approximately 50-fold increase in zero shear viscosity. PG and BG added to Na-CMC_1.2-L_ solutions also cause an increase in zero viscosity. However, in this case, the differences in viscosity between Na-CMC_1.2-L_ solutions containing PG and BG are small, and the increase in their zero viscosity compared to the value of zero shear viscosity obtained for the solution in distilled water is only seven-fold.

**Figure 4:**
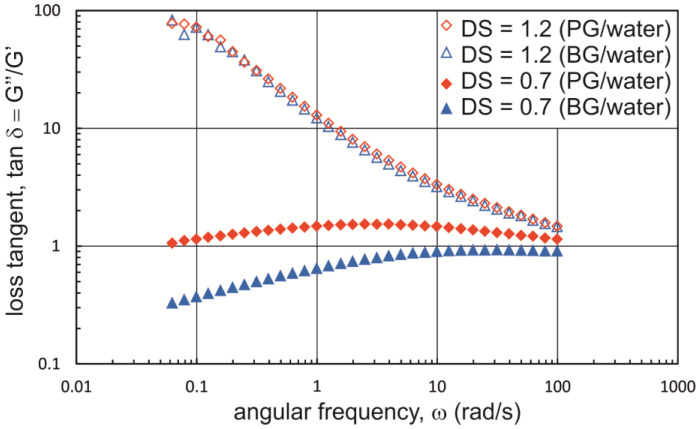
Relationship tan δ = f(ω) for Na-CMC solutions with different degrees of substitution in PG/water and BG/water mixtures (Na-CMC concentration = 2.2%; M_w_ = ~250,000 g·mol^−1^).

The results of oscillation tests shown in Figure 4 point towards a strong effect of the degree of substitution on the viscoelastic properties of Na-CMC solutions in PG/water and BG/water mixtures. These findings are qualitatively consistent with those presented for Na-CMC solutions in a PG/water mixture in our previous paper [22]. The values of the tangent of the phase shift angle (loss tangent, tan δ) are considerably smaller in Na-CMC solutions with a lower degree of substitution (DS = 0.7). In addition, the values of tan δ are lower in Na-CMC_0.7-L_ solutions dissolved in a BG/water mixture. In this case, the tangent of the phase shift angle assumes values less than unity in the entire range of oscillation frequencies, which shows that the solutions have predominantly elastic rather than viscous properties.

A comparison of the rheological properties of Na-CMC solutions in PG/water and BG/water mixtures shows that for DS = 1.2, the effects of the two dihydroxy alcohols used on zero shear viscosity and viscoelastic properties are qualitatively similar, though certain quantitative differences can be detected. Na-CMC solutions with a degree of substitution of 0.7 in a BG mixture exhibit decidedly higher zero shear viscosity, and tan δ has markedly lower values. The variation in zero shear viscosity and the tangent of the phase shift angle may be due to a change in relative coil size and interchain interactions, likely resulting from changes in electrostatic interactions, hydrogen bonding, and hydrophobic interactions [24,42]. According to Lopez et al. [24], the observed rapid viscosity increase in aqueous Na-CMC solutions with low degrees of substitution over the blob overlap concentration results from a large number of interchain hydrophobic associations. The data presented in [39] show that a PG/water mixture is a poorer solvent than pure water, which may be conducive to the association of weakly substituted chains.

### 3.2. Influence of Molecular Weight

**Figure 5:**
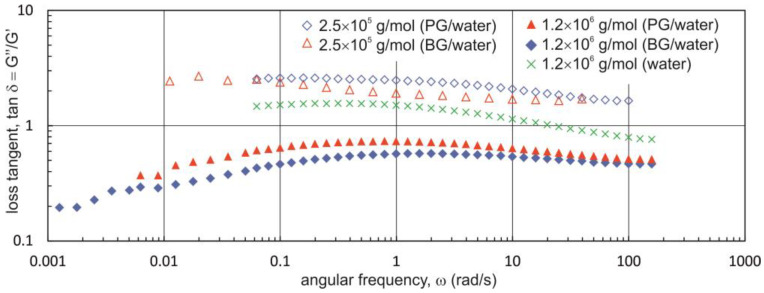
Dependence of the loss tangent versus angular frequency for Na-CMC solutions with different average molecular weights (Na-CMC concentration = 1.6%; PG and BG concentration is 50%).

Figure 5 shows a comparison of the relationship tan δ = f(ω) for the aqueous solutions Na-CMC_0.7-H_ (1.6%) and Na-CMC_0.7-L_ (1.6%) and BG/water and PG/water mixtures. From the data shown in Figure 5, it is evident that in the solutions containing Na-CMC with a higher molecular mass, the tangent of the phase shift angle assumes values less than unity. In addition, similarly to Na-CMC solutions with a molecular mass of 250,000 g/mol, the values of tan δ determined for the solutions containing BG are smaller than those established for the solutions prepared with PG. The results shown in Figure 5 were obtained from tests carried out for mixtures containing 50% PG or 50% BG. Data reported in our previous paper [22] indicate that at a Na-CMC concentration of 1.6%, values of tan δ less than unity could only be observed starting at a PG concentration of 80%.

**Figure 6:**
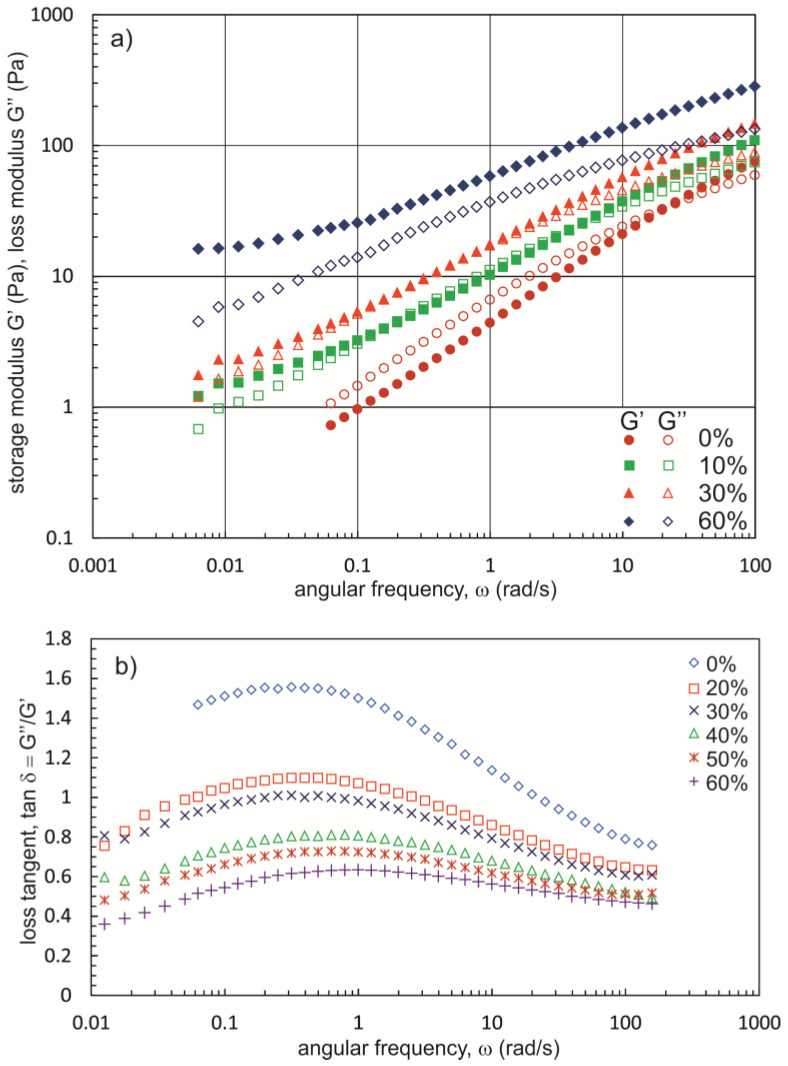
(**a**) Mechanical spectra and (**b**) dependence of the loss tangent versus angular frequency for NaCMC_0.7-H_ (1.6%) solutions with different contents of propane-1,2-diol.

**Figure 7:**
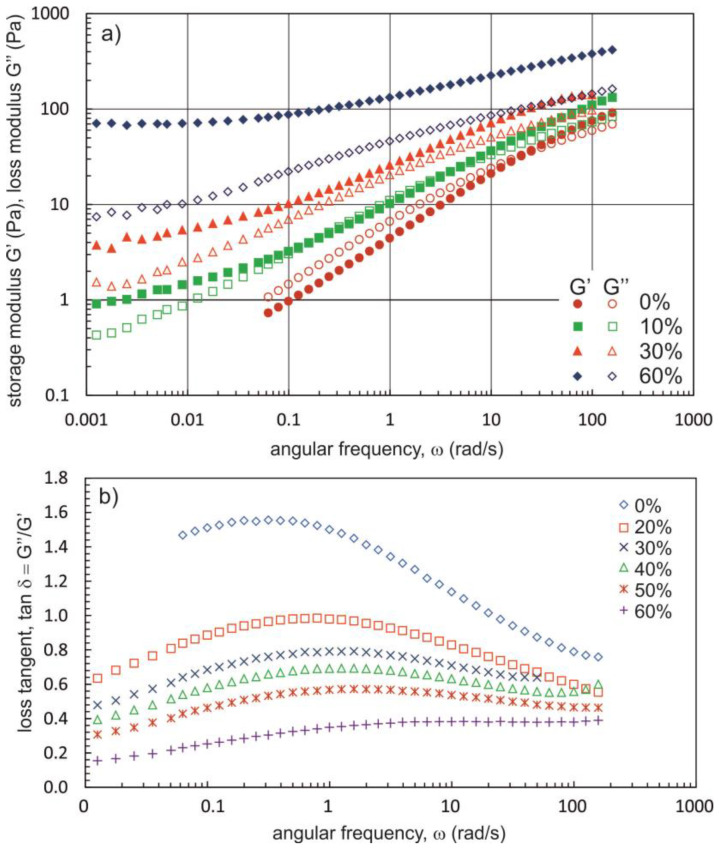
(**a**) Mechanical spectra and (**b**) dependence of the loss tangent versus angular frequency for NaCMC_0.7-H_ (1.6%) solutions with different contents of butano-1,3-diol.

Figure 6 and Figure 7 show the effect of PG and BG concentrations on the shape of mechanical spectra and the relationship tan δ = f(ω). The values of tan δ decrease with increasing concentrations of dihydroxy alcohols, which is evidence for the progressive association of Na-CMC_0.7-H_ chains. In addition, from a PG concentration of 40% and from a BG concentration of 30%, the values of the G′ modulus are greater than those of the G″ modulus (tan δ < 1) over the entire oscillation frequency range. According to the operational definition, a gel is a substance for which tan δ values are less than 0.1 in the oscillation frequency range of 0.001 rad/s to 100 rad/s [47]. Of the solutions for which tests were carried out, the lowest tan δ values were recorded for the solution containing Na-CMC_0.7-H_ at a concentration of 1.6% and BG equal to 60%. The values of the phase shift angle for this fluid range from 0.16 to 0.32. For this type of substance, Ross-Murphy [48] proposed the name ‘weak gel’ or ‘structural fluid’.

The shape of the curves *G*′ = *f*(*ω*) indicates that within the oscillation frequency range of approximately 0.01 rad/s to 100 rad/s, they can be described with the power equation:

(2) $$G^{\prime }=k^{\prime }\cdot \omega^{n^{\prime }}$$

where *k*′ represents constants, and *n*′ is an exponent that gives information about the strength and nature of the gel. Generally, the gel strength increases with a decrease in the *n*′ value. The value *n*′ = 0 is characteristic of covalent gel, *n*′ > 0 indicates a physical gel, and a value of *n*′ close to unity is characteristic of viscous fluids [49,50,51]. The values of the *k*′ and *n*′ parameters for Na-CMC_0.7-H_ solutions in PG/water and BG/water mixtures are listed in Table 2.

The calculated values of *n*′ are characteristic for weak gels [52]. As shown in Table 2, the *n* values decreased with increasing polyhydroxy alcohol concentrations, indicating an increase in the strength of the network structure. Furthermore, at the same concentrations of polyhydroxy alcohol, smaller *n* values were obtained for solutions containing BG, which shows that the addition of this compound to Na-CMC solutions leads to the formation of a stronger network structure than the addition of PG does.

## 4. Conclusions

The analysis of rheological tests shows that it is possible to obtain Na-CMC solutions with diverse properties by introducing dihydroxy alcohols into the formulations. Butane-1,3-diol, propane-1,2-diol, and Na-CMC are used as ingredients in cosmetics and drug carriers. Consequently, the findings reported in this paper may be helpful in designing the rheological properties of pharmaceuticals and personal care products.

The studies show that the molecular mass of the polymer and its degree of substitution have a major effect on the viscoelastic properties of Na-CMC solutions in dihydroxy alcohol/water mixtures. The type of dihydroxy alcohol used also affects the rheological properties of Na-CMC solutions.

If the formulation efforts are oriented towards obtaining a fluid with predominantly viscous rather than elastic characteristics (a viscoelastic fluid), the recommended method is to use Na-CMC with DS ≥ 1. In contrast, if the goal is to obtain a weak gel, Na-CMC with a low degree of substitution is required. Obtaining viscoelastic solutions or weak gels at lower concentrations of dihydroxy alcohols is possible by using Na-CMC with a higher molecular mass. Regardless of the type of dihydroxy alcohol added to Na-CMC solutions, this additive always contributes to an increase in the viscosity of the system, but the fluids obtained differ in their elastic properties. Assuming that the final product can contain both propane-1,2-diol and butane-1,3-diol, the preferred method to obtain a viscoelastic fluid is to use Na-CMC with a high DS and propane-1,2-diol. However, if the goal is to produce a weak gel, the recommended procedure involves using Na-CMC with a higher molecular mass and a low DS and adding butane-1,3-diol to the solution. Our preliminary research shows that Na-CMC solutions in water/PG/BG ternary mixtures also have interesting rheological properties. Our next work will be devoted to this topic

## Figures and Tables

**Table 1:** Characteristics of Na-CMC solutions (BG and PG concentration = 50%; Na-CMC concentration = 2.2%).

DS	Solvent	*η*_0_ (Pa·s)	*λ* (s)	*m*
0.7	Water	1.842	0.168	0.455
0.7	PG/water	451.4	-	-
0.7	BG/water	99.4	-	-
1.2	Water	0.328	0.00234	0.858
1.2	PG/water	2.264	0.0160	0.761
1.2	BG/water	2.427	0.0168	0.763

**Table 2:** Values of parameters *k*′ and *n*′ of Equation (2) for Na-CMC_0.7-H_ (1.6%) solutions in PG/water and BG/water mixtures.

Dihydroxy Alcohol Concentration (%)	*k*’	*n’*	*R* ^2^
PG/water			
10	10.89	0.514	0.998
20	12.50	0.522	0.998
30	17.39	0.489	0.998
40	31.04	0.421	0.998
50	38.85	0.388	0.998
60	59.91	0.345	0.998
BG/water			
10	10.91	0.503	0.997
20	15.74	0.482	0.998
30	26.81	0.389	0.994
40	40.59	0.357	0.996
50	57.66	0.308	0.996
60	141.53	0.200	0.986

## Data Availability

Not applicable.
